# High glucose induces an activated state of partial epithelial-mesenchymal transition in human primary tubular cell cultures

**DOI:** 10.1371/journal.pone.0279655

**Published:** 2023-02-24

**Authors:** Barbara Torsello, Sofia De Marco, Silvia Bombelli, Ingrid Cifola, Ivana Morabito, Lara Invernizzi, Chiara Meregalli, Nicola Zucchini, Guido Strada, Roberto A. Perego, Cristina Bianchi

**Affiliations:** 1 School of Medicine and Surgery, University of Milano-Bicocca, Monza, Italy; 2 Institute for Biomedical Technologies (ITB), National Research Council (CNR), Segrate, Italy; 3 Pathology Unit, Azienda Socio Sanitaria Territoriale (ASST) Monza, San Gerardo Hospital, Monza, Italy; 4 ASST North Milan, Bassini Hospital, Cinisello Balsamo, Italy; Indiana University Purdue University at Indianapolis, UNITED STATES

## Abstract

Tubulointerstitial fibrosis is observed in diabetic nephropathy. It is still debated whether tubular cells, undergoing epithelial-mesenchymal transition (EMT) in high glucose (HG) conditions, may contribute to interstitial fibrosis development. In this study, we investigated the phenotypic and molecular EMT-like changes and the alteration of inflammatory and fibrogenic secretome induced by HG in human primary tubular cell cultures. Taking advantage of this *in vitro* cell model composed of proximal and distal tubular cells, we showed that HG-treated tubular cells acquired a fibroblast-like morphology with increased cytoplasmic stress fibers, maintaining the expression of the epithelial markers specific of proximal and distal tubular cells. HG increased Snail1, miRNA210 and Vimentin mesenchymal markers, decreased N-cadherin expression and migration ability of primary tubular cells, while E-cadherin expression and focal adhesion distribution were not affected. Furthermore, HG treatment of tubular cells altered the inflammatory cytokine secretion creating a secretome able to enhance the proliferation and migration of fibroblasts. Our findings show that HG promotes an activated state of partial EMT in human tubular primary cells and induces a pro-inflammatory and pro-fibrogenic microenvironment, supporting the active role of tubular cells in diabetic nephropathy onset.

## Introduction

Diabetic nephropathy occurs in about 40% of cases of diabetes mellitus and it is the first responsible of end-stage renal disease in the developed world [1]. The chronic exposure to hyperglycemia contributes to the development of the tubulointerstitial changes characterized by thick tubular basement membrane, tubule atrophy and interstitial fibrosis and observed in overt diabetic nephropathy [2]. A direct involvement of tubular cells has been suggested in diabetic nephropathy establishment. Some studies have shown that tubular cells treated with high glucose concentrations (hereafter, HG-treated) undergo an *in vitro* epithelial-mesenchymal transition (EMT) that might have a direct role in tubulointerstitial fibrosis development [3–5]. The EMT induced by HG treatment of tubular epithelial cell lines is characterized in vitro by the up-regulation of N-cadherin, Vimentin, α-SMA and Snail, assessed in tubular cell lines as mesenchymal markers, and the down-regulation of E-cadherin and ZO-1, assessed as epithelial markers [6–8]. During EMT, epithelial cells undergo phenotypic changes, due to the loss of epithelial markers and over-expression of mesenchymal genes, characterized by myofibroblast transformation and consequent accumulation of extracellular matrix [9]. However, the origin of myofibroblasts in diabetic nephropathy fibrosis is still in debate [10] and the existence of a full EMT process *in vivo* is under debate. Some papers provide evidence that human and rodent tubular cells through EMT give rise to matrix-producing interstitial fibroblasts [11–13]. In other papers mouse tubular cells produced collagen without evidence of EMT [14,15]. However, some other researches by lineage-tracing approaches have identified endogenous stromal cells (fibroblasts and pericytes) as the primary source of collagen [16,17]. Instead, the tubular epithelium undergoing EMT was described as a possible source of collagen only in advanced stages of the disease, when the basement membrane has suffered severe damages [18].

Moreover, it has been described that, in response to noxious stimuli like HG, tubular renal cells secrete pro-inflammatory and pro-fibrotic molecules that promote the recruitment of inflammatory cells with a positive feedback mechanism. This inflammatory state may lead to EMT of the tubular cells, recruitment of fibrocytes and proliferation/differentiation of fibroblasts to myofibroblasts, all mechanisms contributing to fibrosis progression [2]. Notably, tumor necrosis factor α (TNFα), IL-1 and IL-6 are increased in serum and urine of patients affected by diabetic nephropathy and are associated with the progression of renal injury [1]. Different renal cell types in HG conditions produce MCP-1, the key mediator of renal infiltration and recruitment of monocytes that are also responsible for pro-inflammatory and pro-fibrotic cytokine release [9]. Moreover, HG-dependent activation of inflammasome in HK-2 human proximal tubular cell line has been described to induce the increase of IL-1b and IL-18 secretion [19].

It has been also described that proximal tubular cell lines of human, mouse and rat origin in HG conditions increase their *in vitro* production of TGF-β [6,8,20], a well-known fibrogenic cytokine involved in the development of renal interstitial fibrosis [21].

The aim of our study was to evaluate, by immunophenotypical, molecular and functional assays, the EMT-like changes induced by HG treatment in human primary tubular cell cultures established from normal cortex. Taking advantage of this *in vitro* cell model, we also assessed the changes induced by HG treatment in tubular cell secretome and involved in fibroblast functional response.

## Materials and methods

### Establishment of human primary tubular cell cultures and treatments

Primary tubular cell cultures were obtained from 44 independent human renal cortex tissue specimens and characterized as previously described [22–27]. Normal renal cortex specimens were obtained from adult human kidneys surgically removed because of renal carcinoma, after written patients’ informed consent and accordingly with the Declaration of Helsinki recommendations and with Ethical Committee “Comitato Etico Azienda Ospedaliera San Gerardo” (No. 1532, 17 November 2011). At the first confluence, the cells were detached with trypsin and replated to reach the second confluence at the end of each treatment period. After 24h of serum starvation, the cells were cultured in low glucose Dulbecco’s modified Eagle’s medium (DMEM) (100 mg/dl glucose; control medium), or in high glucose DMEM (450 mg/dl glucose; HG medium), both supplemented with 10% fetal bovine serum (FBS), 1% glutamine, 1% penicillin-streptomycin and 1% amphotericin (Euroclone, Milan, Italy) for up to 7 days. The glucose concentration was regularly checked with Atellica CH Glucose Hexokinase_3 (GluH_3) assay by Atellica Solution Analyzer (Siemens, Munich, Germany), and, when necessary, restored by addition of fresh D-glucose (Sigma-Aldrich, St Louis, MO, USA). Osmolality balance was obtained by addition of D-mannitol (350 mg/dl; Sigma-Aldrich) in control medium.

### Immunofluorescence and stress fiber analysis

Cells were seeded on glass coverslips, fixed with 4% PFA (Sigma-Aldrich) and incubated overnight at 4°C with the following primary antibodies: anti-PanCytokeratin (Mouse 1:200, Dako, Glostrup, Denmark), anti-Epcam (Mouse 1:1000, clone HEA-125, GeneTex, Irvine, CA, USA), anti-AQ-1 (Mouse 1:50, clone B-11, Santa Cruz Biotechnology, Heidelberg, Germany), anti-CD13-PE (Mouse 1:25, Biolegend, San Diego, CA, USA), anti-CD13-FITC (Mouse 1:25, Abcam, Cambridge, UK), anti-N-cadherin (Rabbit 1:50, Abcam, and Mouse 1:50, clone 32/N, Becton Dickinson, San Josè, CA, USA), anti-Calbindin (Mouse 1:100, clone CB-955, Sigma-Aldrich), anti-E-cadherin (Mouse 1:50, Becton Dickinson, and Rabbit 1:50, Cell Signalling Technology, Danvers, MA, USA) and anti-Paxillin (Mouse 1:50, Becton Dickinson). When necessary, the secondary antibodies Alexa 488 conjugated anti-mouse IgG and Alexa 594 conjugated anti-rabbit IgG (1:100, Molecular Probes, Carlsberg, CA, USA) were used. Stress fibers were labeled by Alexa-Fluor-594-phalloidin (1:100, Molecular Probes) and nuclei counterstained with Mounting DAPI (Molecular Probes). Immunofluorescence images were obtained with a Zeiss LSM810 confocal microscope, using a 63x objective, equipped with Zen2009 software (Zeiss, Oberkochen, Germany).

### Evaluation of tubular cell migration

For wound healing assay, monolayers of human tubular primary cells were plated on 6-well plates and cultured for 24h, 96h and 7 days in control and HG medium. At each time point, the monolayers were scratched with a pipette tip and photographed with a digital camera mounted on an Olympus inverted microscope (100x magnification, Olympus Life Science, Tokyo, Japan). The cultures were photographed again after 8h. To quantify wound recovery, initial and final wound width was measured using *segmented lines* tool of ImageJ software (National Institute of Health, Bethesda, MD, USA) to track two segmented lines corresponding to the wound edges, as previously described [28].

For Boyden chamber assay, 2x10^4^ cells grown in control or HG medium for the indicated time points were seeded in the upper chamber of a 8-μm transwell membrane (Corning Costar Corp, Cambridge, MA, USA) and, after 6h at 37°C, cells adherent to the lower membrane were fixed with cold methanol for 15 min, stained with Haematoxylin-Eosin solution (Sigma-Aldrich) and microphotographed. The migrated cells were counted with ImageJ software, as previously described [25]. Two membranes were analyzed for each sample.

### RNA extraction and real-time quantitative PCR

Total RNA extraction and reverse transcription were carried out as previously described [29]. Real-time quantitative PCR assays were carried out in duplicate for each sample, with the following TaqMan Gene Expression Assays: E-Cadherin (Hs01023894_m1), Col1a2 (Hs00164099_m1), TGF-β1 (Hs00998133_m1), Snail1 (Hs00195591_m1), Zeb2 (Hs00207691_m1), Fibronectin (Hs01549976_m1) and GAPDH (Hs99998805_m1) as endogenous control (all Applied Biosystems, Foster City, CA, USA), according to manufacturer’s instructions, using an ABI PRISM® 7900HT Fast Real-Time PCR System (Applied Biosystems). For microRNA quantification, a TaqMan microRNA assay (Applied Biosystems) was used. Briefly, 10 ng of total RNA were retro-transcribed in 20 μl total volume reaction containing 3 μl of 5x miRNA specific primers (RT 2300 miRNA145, miRNA210, miRNA149 and RNU48 as endogenous control), 19 μl of 20 U/μl RNase inhibitor, 0.15 μl of 100 mM dNTPs and 1 μl of 50 U/μl Multiscribe Reverse Retrotranscriptase (Applied Biosystems). The reverse transcription conditions were: 16°C for 30 min, 42°C for 30 min and 85°C for 5 min. Then, 0.5 ng of the specific cDNA obtained was amplified in 15 μl total volume reaction containing 10 μl of TaqMan Universal Master Mix II no UNG and 1 μl of specific primers and probes (Applied Biosystems). The PCR reaction was performed in duplicate for each sample as follows: 95°C for 10 min, 40 cycles at 95°C for 15 s and 60°C for 1 min. The relative expression levels of the different targets, expressed as 2^−ΔΔCt^, were represented as fold change with respect to control samples considered equal to 1.

### Protein extraction and western blotting

Primary tubular cell cultures were lysed at the indicated time points. 30 μg of protein lysates were quantified with a BCA microassay (Sigma-Aldrich), separated on NuPage 4–12% gels (Invitrogen, Carlsbad, CA, USA) and submitted to western blotting [24] with the following antibodies: anti-N-cadherin (Mouse 1:1000, clone 32/N, Becton Dickinson), anti-E-cadherin (Mouse 1:100, clone 36, Becton Dickinson), anti-Vimentin (Mouse 1:1000, Dako), anti-α-SMA (Rabbit 1:1000, Dako), anti-TGF-β (Rabbit 1:1000, Cell Signaling Technology), anti-β-Actin (Rabbit 1:1000, Sigma-Aldrich). Densitometric analysis of specific bands was performed by ImageJ software, and for quantification the specific band intensities were normalized to the corresponding β-Actin band intensity.

### Quantification of cytokines secreted in conditioned media

Conditioned culture media of tubular cells were used for the analysis of secreted human chemokines, cytokines and growth factors by using the Bio-Plex Pro Human Cytokine, Chemokine and Growth Factor Assays (Human Group I and Human Group II Panels, Bio-Rad Laboratories, Hercules, CA, USA), coupled with BioPlex Luminex platform, following the manufacturer’s instructions. Culture media from primary tubular cell cultures growth in control and HG medium were collected at different time points and analyzed by Luminex platform. Cytokine concentration values were normalized for the cell culture protein content and expressed as pg/mg. Cytokine secretion kinetics were assessed in duplicate in two independent experiments.

Functional classification for cytokines of interest was performed by annotating the corresponding genes for associated Gene Ontology Biological Process (GO-BP) terms, using The Database for Annotation, Visualization and Integrated Discovery (DAVID) tool (https://david.ncifcrf.gov/) and ToppGene suite (https://toppgene.cchmc.org/) as functional annotation tools.

### Secreted TGF-β1 quantification by ELISA

Conditioned culture media of tubular primary cell cultures grown for 96h in control and HG medium were used to quantify secreted TGF-β1 by using Human TGF-β1 Platinum ELISA kit (Affymetrix eBioscience, Vienna, Austria), according to the manufacturer’s instructions. Absorbance at 450 nm was measured using an automated microplate reader (Victor Wolla C1420, Perkin Elmer, Woltham, MA, USA). Data were normalized for the cell culture protein content and expressed as pg/mg.

### Evaluation of NIH3T3 fibroblast growth and migration

The NIH3T3 mouse fibroblast cell line was cultured in conditioned medium obtained from primary tubular cell cultures grown for 96h in control and HG medium. Cells were counted after 24h and 48h using Trypan Blue solution 0.4% (Sigma-Aldrich) with Thoma chamber. NIH3T3 cells were seeded in 6-well plate and cultured for 24h in control and HG conditioned media obtained from primary tubular cell cultures. After 24h, the NIH3T3 monolayers were scratched with a pipette tip to perform the wound healing assay as previously described.

### Statistical analysis

All molecular and functional effects were evaluated and/or quantified by two different operators blinded to experimental treatment. Differences between two groups were analysed by paired t-test, using OriginPro 2016 64BIT software (Origin Lab Corporation, Northampton, MA, USA). P-values <0.05 were considered as statistically significant. In the box/dot graphs, each dot represents one single independent experiment, the boxes indicate the 25°-75° percentile, the continuous horizontal line into the box represents the mean, while the dotted horizontal line represents the median. Maximum and minimum values are also indicated.

## Results

### Phenotypical characterization of HG-treated primary tubular cell cultures

**Fig 1:**
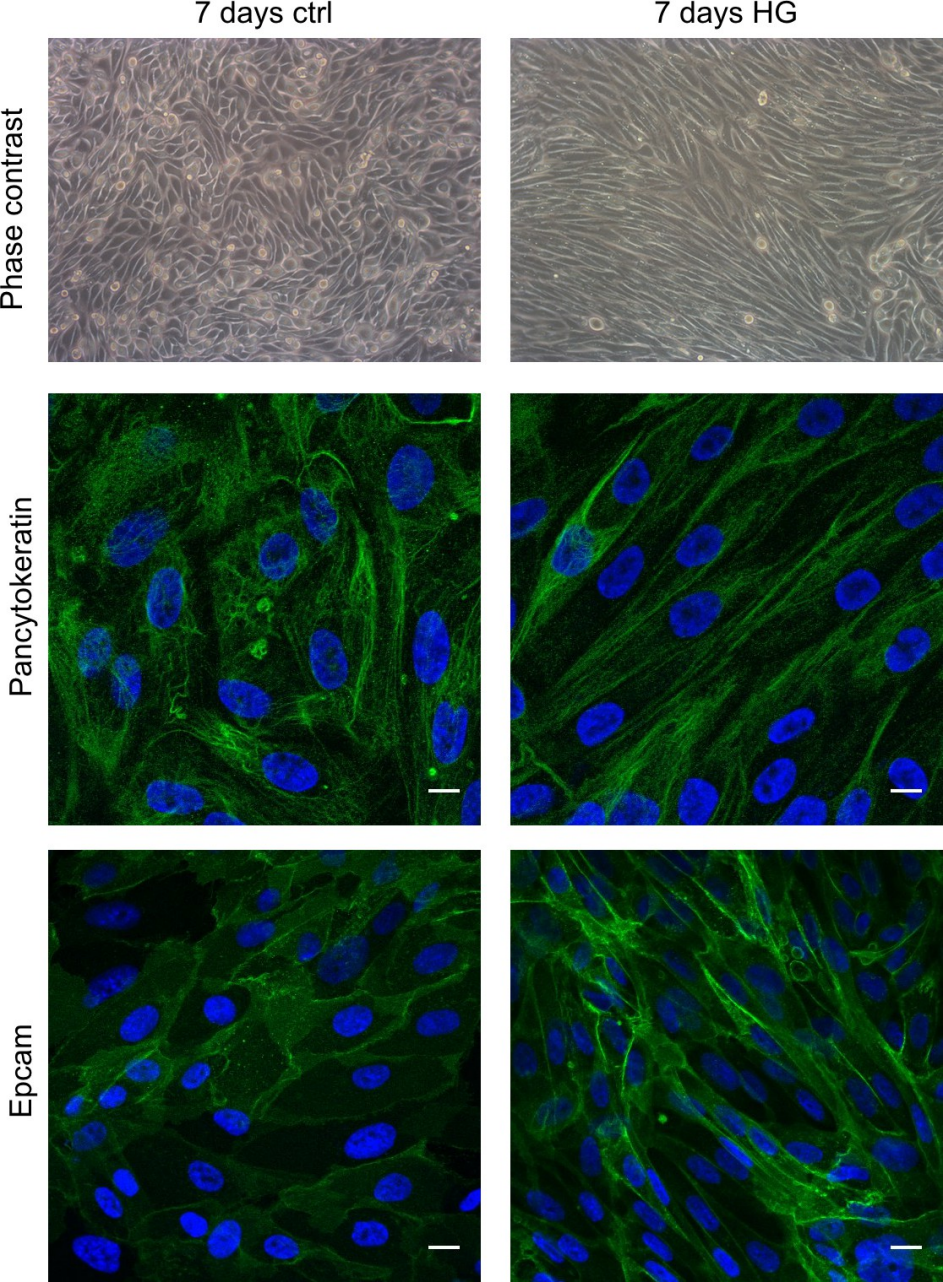
Representative morphological evaluation and cell distribution of epithelial markers in primary tubular cell cultures grown for 7 days in control (ctrl) or high glucose (HG) medium. The phase contrast images (200x) show the different cell morphology. The confocal microscopy images show the cell distribution of the epithelial markers Pancytokeratin and Epcam (green). DAPI (blue) was used to counterstain the nuclei. Scale bars: 10 μm.

**Fig 2:**
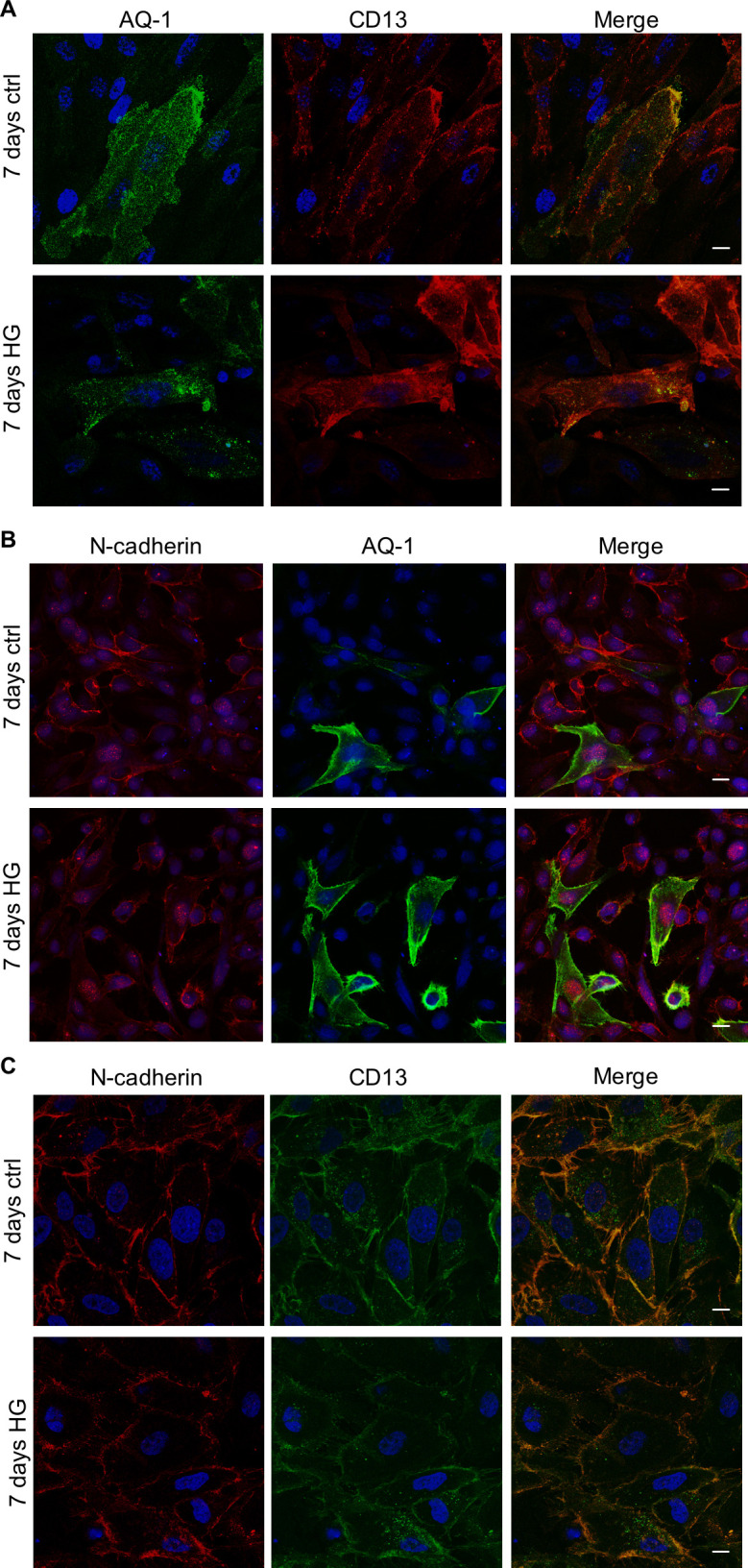
Cell distribution of proximal tubular markers in primary tubular cell cultures grown for 7 days in control (ctrl) or high glucose (HG) medium. Representative confocal microscopy images of cell cultures stained with antibodies against: (**A**) Aquaporin-1 (AQ-1, green) and CD13 (red); (**B**) N-cadherin (red) and AQ-1 (green); (**C**) N-cadherin (red) and CD13 (green). The merge panels show the co-localization signals. DAPI (blue) was used to counterstain the nuclei. Scale bars: 10 μm.

**Fig 3:**
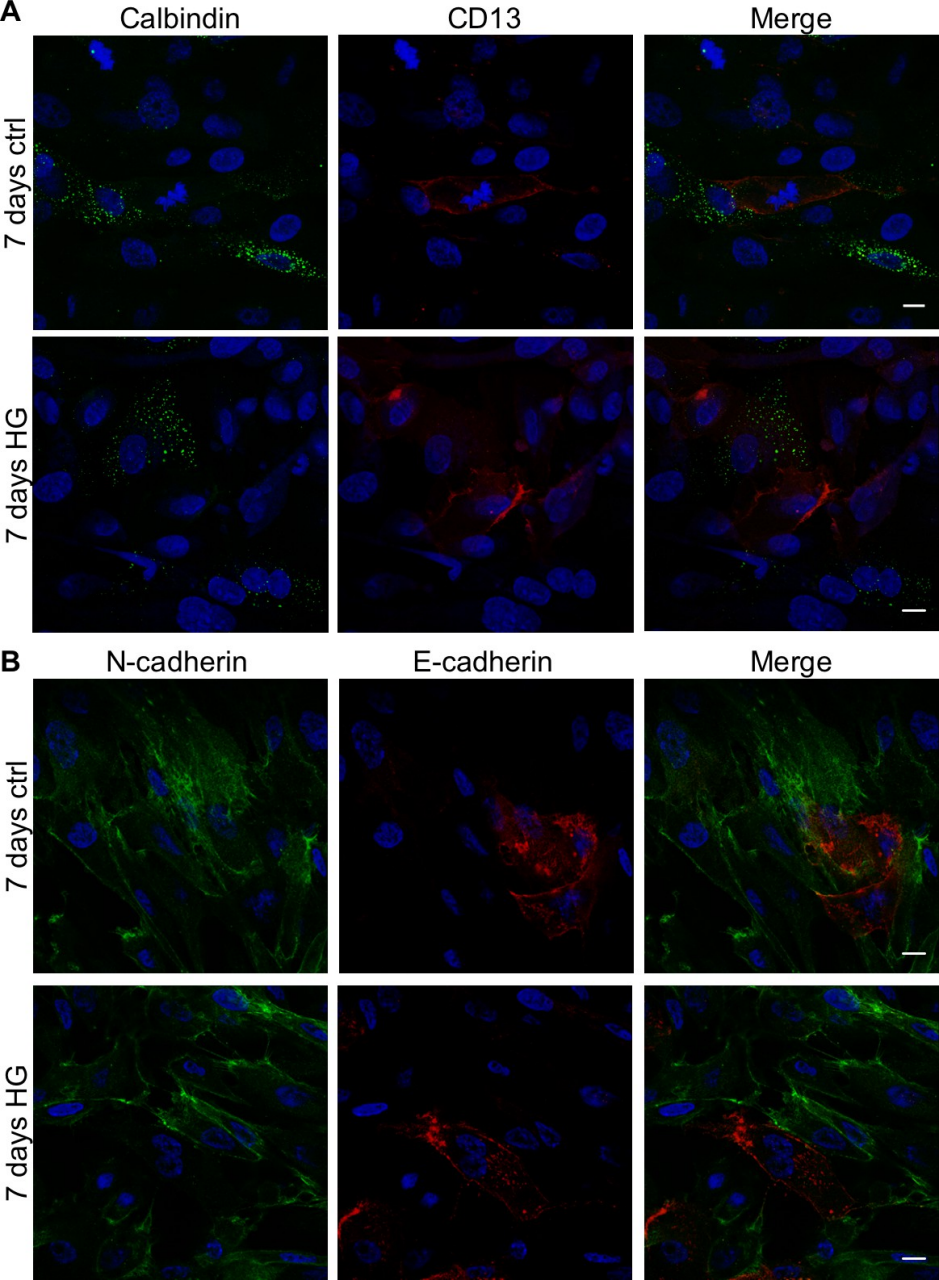
Cell distribution of proximal and distal tubular markers in primary tubular cell cultures grown for 7 days in control (ctrl) or high glucose (HG) medium. Representative confocal microscopy images of cell cultures stained with antibodies against: (**A**) Calbindin (green) and CD13 (red); (**B**) N-cadherin (green) and E-cadherin (red). The merge panels show the co-localization signals. DAPI (blue) was used to counterstain the nuclei. Scale bars: 10 μm.

We treated human primary tubular cell cultures for 7 days with HG medium, which did not significantly change cell viability as documented by AnnexinV/propidium iodide FACS analysis (S1A Fig), and we evaluated the immunophenotypical changes induced by this treatment. By phase contrast microscopy, we observed that the cobblestone primary tubular cells grown in the control medium showed a more elongated fibroblast-like morphology when cultured in HG condition (Fig 1). By immunofluorescence (Fig 1) and FACS (S1B Fig) analysis we evidenced that the epithelial markers Pancytokeratin and Epcam were present in control and HG-treated cells and expressed in more than 98% of both control and HG-treated cells. Moreover, the proximal tubular markers AQ-1, CD13 and N-cadherin co-localized in the cells of both control and HG-treated cultures (Fig 2A and 2B). In particular, N-cadherin and CD13 co-localized on the membranes of these cells (Fig 2C). Otherwise, the distal tubular marker Calbindin was not co-expressed with the proximal marker CD13 (Fig 3A). Moreover, both control and HG-treated tubular cells did not co-express the markers of human proximal and distal tubular cells, N- and E-cadherin (Fig 3B). Overall, this immunophenotypical analysis showed that HG treatment induced a fibroblast-like morphology in human primary tubular cells without affecting the cell distribution of the epithelial and specific proximal and distal tubular markers.

### Stress fiber, cell adhesion and migration analysis of human primary tubular cells treated with high glucose

**Fig 4:**
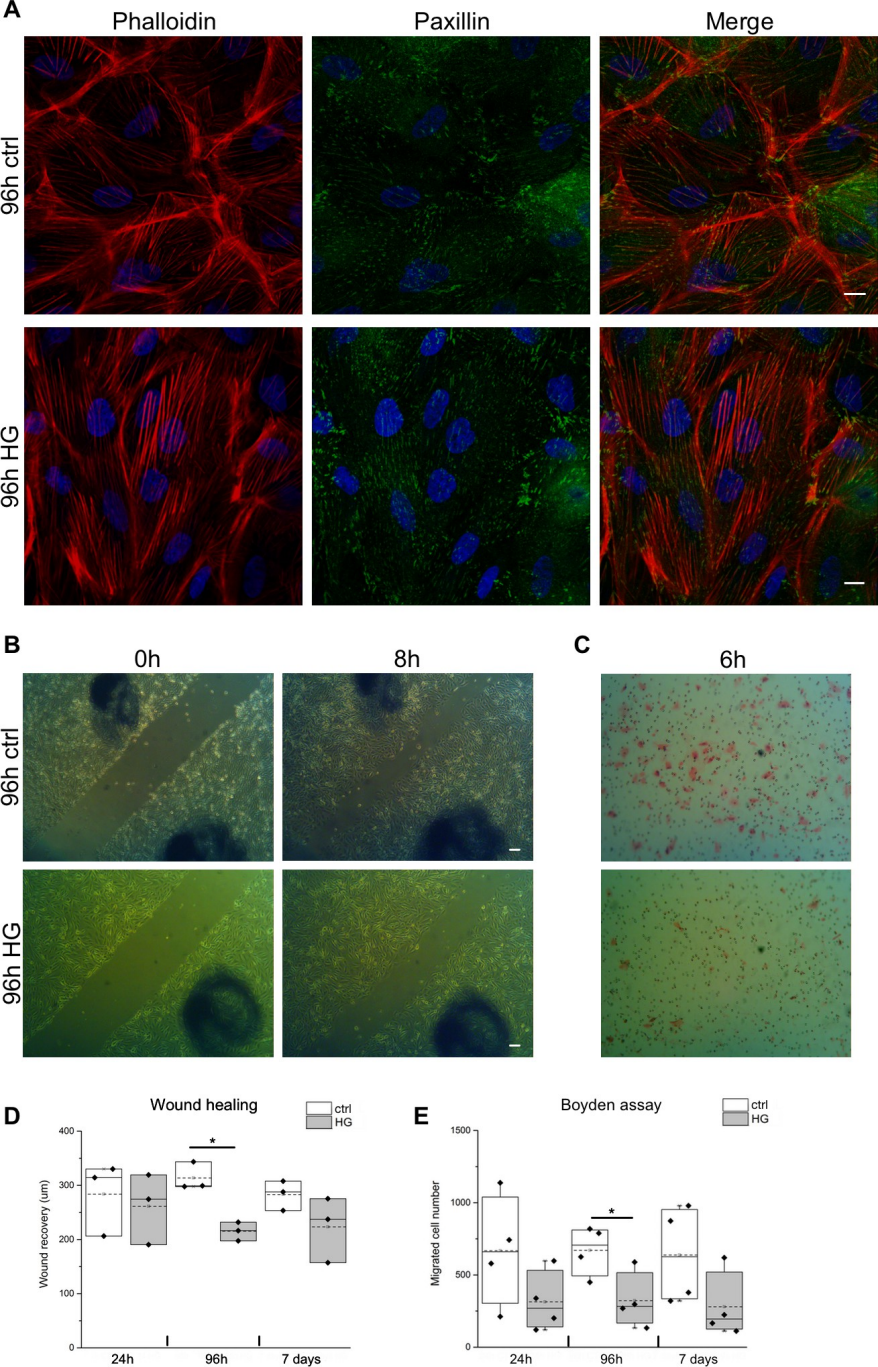
Stress fibers and focal adhesion cellular distribution and migration evaluated in primary tubular cell cultures grown for 96h in control (ctrl) or high glucose (HG) medium. (**A**) Representative confocal microscopy images of cell cultures stained with Alexa-Fluor-594-phalloidin (red) and anti-Paxillin antibody (green). DAPI (blue) was used to counterstain the nuclei. Scale bars: 10 μm. (**B**) Representative phase contrast images of wound healing assay at the scratch (0h) and after 8h of wound recovering performed in primary tubular cultures grown for 96h in control (ctrl) and high glucose (HG) medium. Scale bars: 100 μm. (**C**) Representative images of Haematoxylin-Eosin stained tubular cells grown in control (ctrl) and high glucose (HG) medium for 96h and migrated in Boyden chamber for 6h. (**D**) Wound healing graph representing the recovery expressed as μm in three independent experiments. (**E**) Boyden assay graph reporting the mean number of migrating cells counted in 10 randomly chosen fields for each sample in four independent experiments. *p<0.05.

Epithelial cells undergoing EMT process acquire prominent cytoplasmic stress fibers, increase their motility and decrease their adhesion to substrate [30]. Thus, we evaluated whether HG treatment of our primary tubular cell cultures induced changes in these cytoskeletal and functional features. By immunofluorescence analysis, we evidenced that HG-treated tubular cells showed thick stress fibers crossing the cytoplasm, whereas in control condition the cells were characterized by thinner stress fibers showing a prominent cortical distribution (Fig 4A). The quantitative analysis of stress fiber density performed in HG-treated and control primary cultures (S1C Fig) confirmed that HG induced a significant increment of cytoplasmic stress fibers density in tubular cells. The staining with anti-Paxillin antibody, a marker of focal adhesions, did not evidence significant changes in focal adhesion distribution between control and HG-treated cells. No significant effect of HG on tubular cell adherence to substrate was evidenced by cell adhesion assay (S1D Fig). Moreover, by wound healing assay, we observed that the wound recovery was significantly slower in 96h HG-treated cultures compared to control cultures (Fig 4B and 4D). Boyden chamber assay confirmed that 96h HG-treated cells migrated slower than control ones (Fig 4C and 4E). Thus, our results indicated that HG treatment induced cytoskeletal changes in human primary tubular cells without functional effects consistent with an EMT phenotype.

### Evaluation of EMT marker expression in human tubular primary cell cultures treated with high glucose

**Fig 5:**
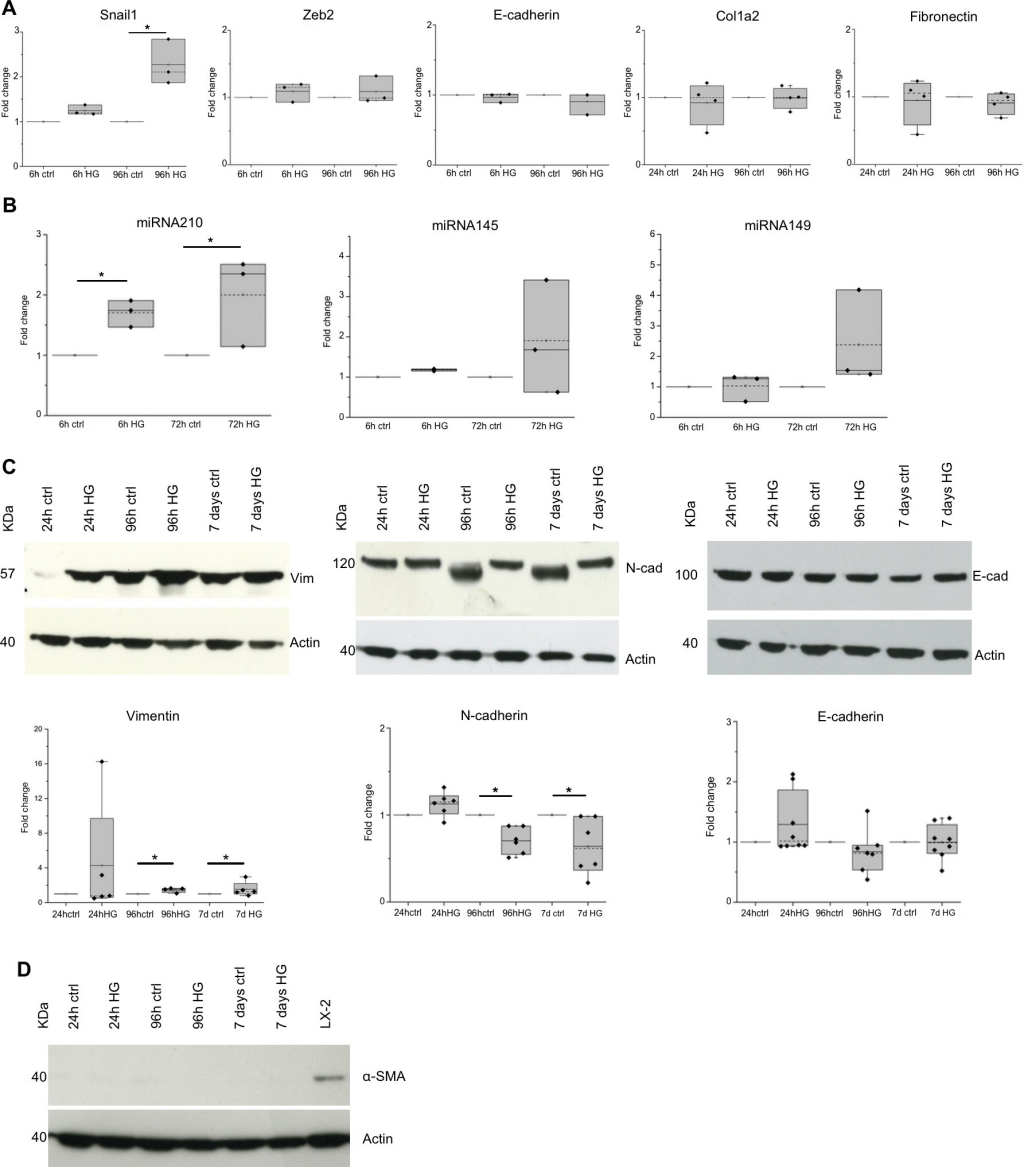
EMT marker expression in primary tubular cell cultures grown in control (ctrl) and high glucose (HG) medium. (**A**) Snail1, Zeb2, E-cadherin, Col1a2, and Fibronectin gene expression and (**B**) miRNA210, miRNA145 and miRNA149 expression evaluated by Real-time PCR at the indicated time points in control (ctrl) and high glucose (HG) conditions. The relative amounts of the different transcripts and miRNAs, calculated as 2^−ΔΔCt^, are expressed as fold change with respect to the corresponding control sample equal to 1. (n≥3). *p<0.05. (**C**) Representative western blot of Vimentin, N-cadherin, E-cadherin and β-actin proteins evaluated in lysates of primary tubular cell cultures grown for the indicated time points in control (ctrl) and high glucose (HG) medium. In the box plots the densitometric values of the specific protein bands normalised for corresponding β-actin bands are expressed as a fold change with respect to corresponding control samples. (n≥4). *p<0.05. (**D**) Representative western blot of α-SMA protein evaluated in lysates of primary tubular cell cultures grown for the indicated time points in control (ctrl) and high glucose (HG) medium. The lysate of LX-2 human fibroblast cell line is used as α-SMA positive control.

It is known that epithelial cells undergoing EMT process show a down-regulation of epithelial markers and an up-regulation of mesenchymal markers [31]. Thus, we evaluated the effects of HG treatment on the expression of different EMT markers in our human tubular primary cell cultures. Real-time quantitative PCR analysis showed that the EMT marker Snail1, but not Zeb2, was significantly up-regulated in 96h HG-treated cells compared to control cells. On the contrary, the gene expression of the tubular epithelial marker E-cadherin as well as the mesenchymal markers Col1a2 and Fibronectin did not significantly change between control and HG-treated cells (Fig 5A). Furthermore, we evaluated the expression of some miRNAs, such as miRNA210, miRNA145 and miRNA149, known to be involved in EMT process [32–35]. In particular, we evidenced that miRNA210 significantly increased after HG treatment (Fig 5B). Even miRNA145 and miRNA149 increased but not in a statistically significant manner. At protein level, the expression of the mesenchymal marker Vimentin, evaluated by western blot, increased after 96h and 7 days of HG treatment, while the expression of the mesenchymal marker α-SMA was undetectable in both control and HG-treated cells along the 7 days of treatment (Fig 5C and 5D). The expression of the distal tubular marker E-cadherin did not change during HG treatment, whereas the expression of the tubular proximal marker N-cadherin significantly decreased in HG-treated cells at 96h and 7 days of treatment (Fig 5C). Given that the percentage of CD13^+^ proximal tubular cells did not significantly change along HG treatment (S1E Fig), we excluded that the decrease of the expression level of N-cadherin protein observed in HG-treated cultures was due to an outgrowth of distal versus proximal tubular cells.

Overall, these data showed that HG treatment induced only a partial EMT phenotype in human primary tubular cell cultures.

### High glucose induces changes in inflammatory and fibrogenic cytokine secretion of human tubular primary cell cultures that enhance fibroblast proliferation and migration

**Fig 6:**
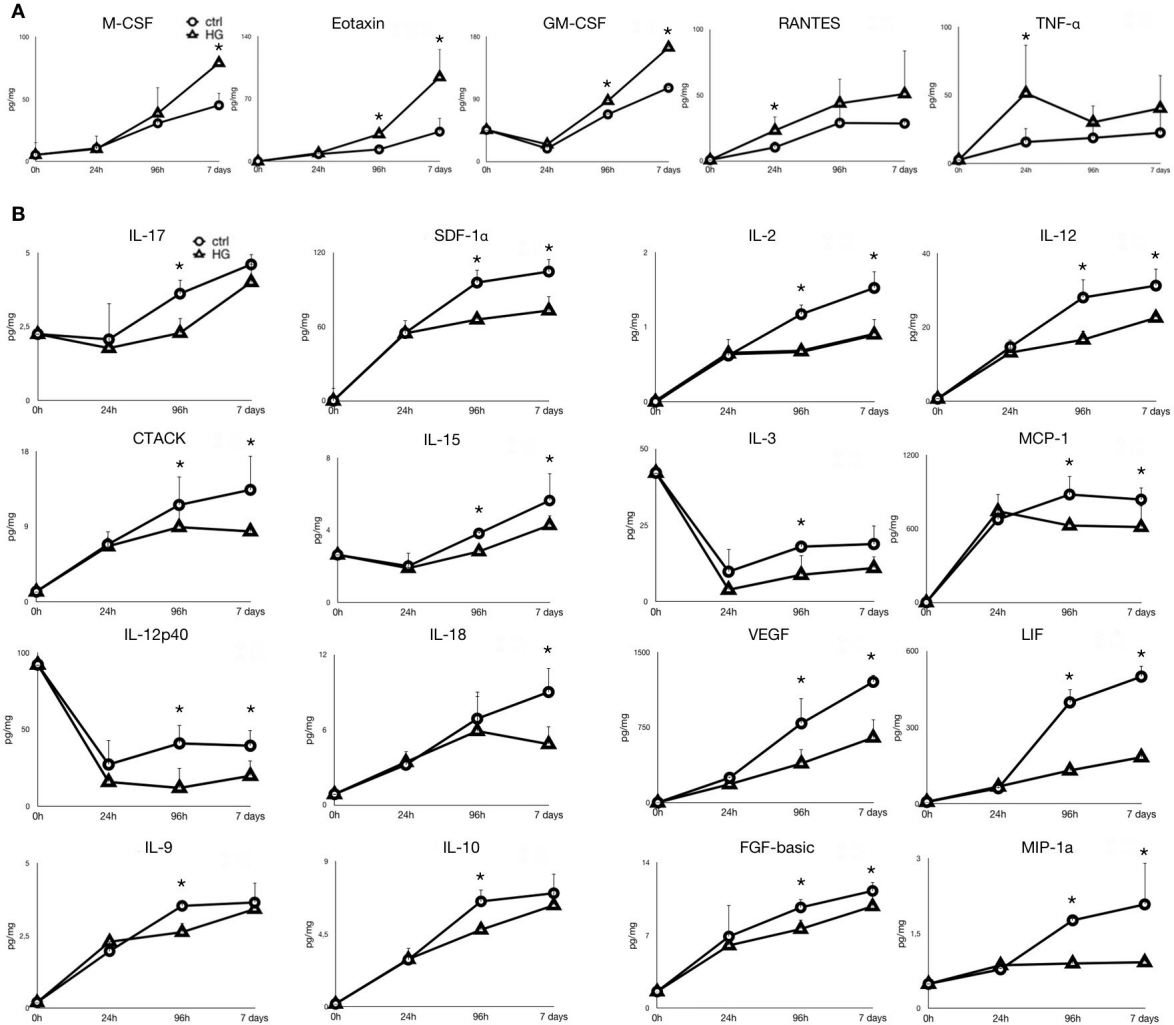
Cytokines, chemokines and growth factors differently secreted by primary tubular cell cultures grown for the indicated time points in control (ctrl) and high glucose (HG) medium. Cytokines increased (**A**) or decreased (**B**) in HG conditioned medium of primary tubular cell cultures compared to control. The plots show the mean concentration of the cytokines, expressed as pg/mg, in control conditioned medium (white circles; ctrl) and HG conditioned medium (white triangles; HG) of two independent experiments analyzed in duplicate. *p<0.05.

Growing evidence supports the notion that inflammation plays an important role in the pathogenesis of diabetic nephropathy and renal fibrosis [36,37]. To investigate whether renal tubular cells can contribute to develop a pro-inflammatory and pro-fibrogenic microenvironment in HG conditions, we quantified cytokines and growth factors secreted in culture media by control and HG-treated tubular cells using a microsphere-based multiplex immunoassay. Our data showed that the secretion of M-CSF, Eotaxin, GM-CSF was significantly increased at 96h and 7 days of HG treatment compare to controls, whereas RANTES and TNF-α secretion already significantly increased at 24h of HG treatment (Fig 6A). The secretion of IL-17, SDF-1α, IL-2, IL-12, CTACK, IL-15, MCP-1, IL-18, VEGF, LIF, IL-9, IL-10, FGF-basic and MIP-1a increased significantly less in HG-treated cultures compared to control. Instead, the decrement of IL-3 and IL-12p40 secretion was significantly more important in HG-treated compared to control cultures (Fig 6B).

To investigate the biological activity of such differential cytokines, we performed a functional annotation analysis for the Gene Ontology Biological Process (GO-BP) terms associated with the corresponding genes, and then we focused on the annotations related to inflammatory and fibroblastic response in order to evaluate whether the secretome of HG-treated tubular cells may support the development of a pro-inflammatory and pro-fibrogenic tubular microenvironment. Globally, we found that four out of the five cytokines significantly increased in media of HG-treated tubular cultures compared to control (M-CSF, Eotaxin, RANTES, TNF-α) were annotated in the “inflammatory response” biological process. Notably, RANTES and TNF-α that significantly increased already at 24h of HG treatment were annotated in GO-BP terms related to “positive regulation of inflammation” (Table 1). On the other hand, nine of the 16 cytokines whose secretion was significantly lower in HG-treated cultures compared to control (IL-17, IL-2, IL-15, MCP-1, IL-12p40, IL-18, IL-9, IL-10, MIP-1a) were annotated in the “inflammatory response” process, and, interestingly, three of them (IL-12p40, IL-10 and IL-2) were associated with a “negative regulation of inflammatory response” function (Table 1). Taken as a whole, these data are in agreement with the hypothesis that HG treatment induced the secretion of pro-inflammatory cytokines and reduced the secretion of anti-inflammatory cytokines by tubular cultures.

**Table 1:** Cytokines up- and down-regulated in HG-treated tubular cell media and annotated in Gene Ontology Biological Process (GO-BP) terms related to inflammatory and fibroblastic response.

UP-regulated cytokines	
*Annotation for GO-BP terms*	*Cytokines*
inflammatory response (GO:0006954)	RANTES,Eotaxin,TNF-α,M-CSF
positive regulation of inflammatory response (GO:0050729); positive regulation of acute inflammatory response (GO:0002675)	RANTES,TNF-α
positive regulation of chronic inflammatory response to antigenic stimulus (GO:0002876); positive regulation of chronic inflammatory response (GO:0002678); positive regulation of inflammatory response to antigenic stimulus (GO:0002863)	TNF-α
DOWN-regulated cytokines	
*Annotation for GO-BP terms*	*Cytokines*
inflammatory response (GO:0006954)	IL-10,IL-2,IL-12p40,MCP-1,MIP-1a,IL-15,IL-17,IL-18,IL-9
regulation of inflammatory response (GO:0050727)	IL-10,IL-2,IL-12p40,MIP-1a,IL-15,IL-18
negative regulation of inflammatory response (GO:0050728)	IL-10,IL-2,IL-12p40
negative regulation of inflammatory response to antigenic stimulus (GO:0002862)	IL-10,IL-12p40
negative regulation of chronic inflammatory response (GO:0002677); negative regulation of chronic inflammatory response to antigenic stimulus (GO:0002875)	IL-10
negative regulation of fibroblast migration (GO:0010764)	FGF- basic

Furthermore, FGF-basic growth factor, whose secretion was lower in HG-treated cultures than in control cultures, was annotated in the “negative regulation of fibroblast migration” GO-BP term (Table 1).

**Fig 7:**
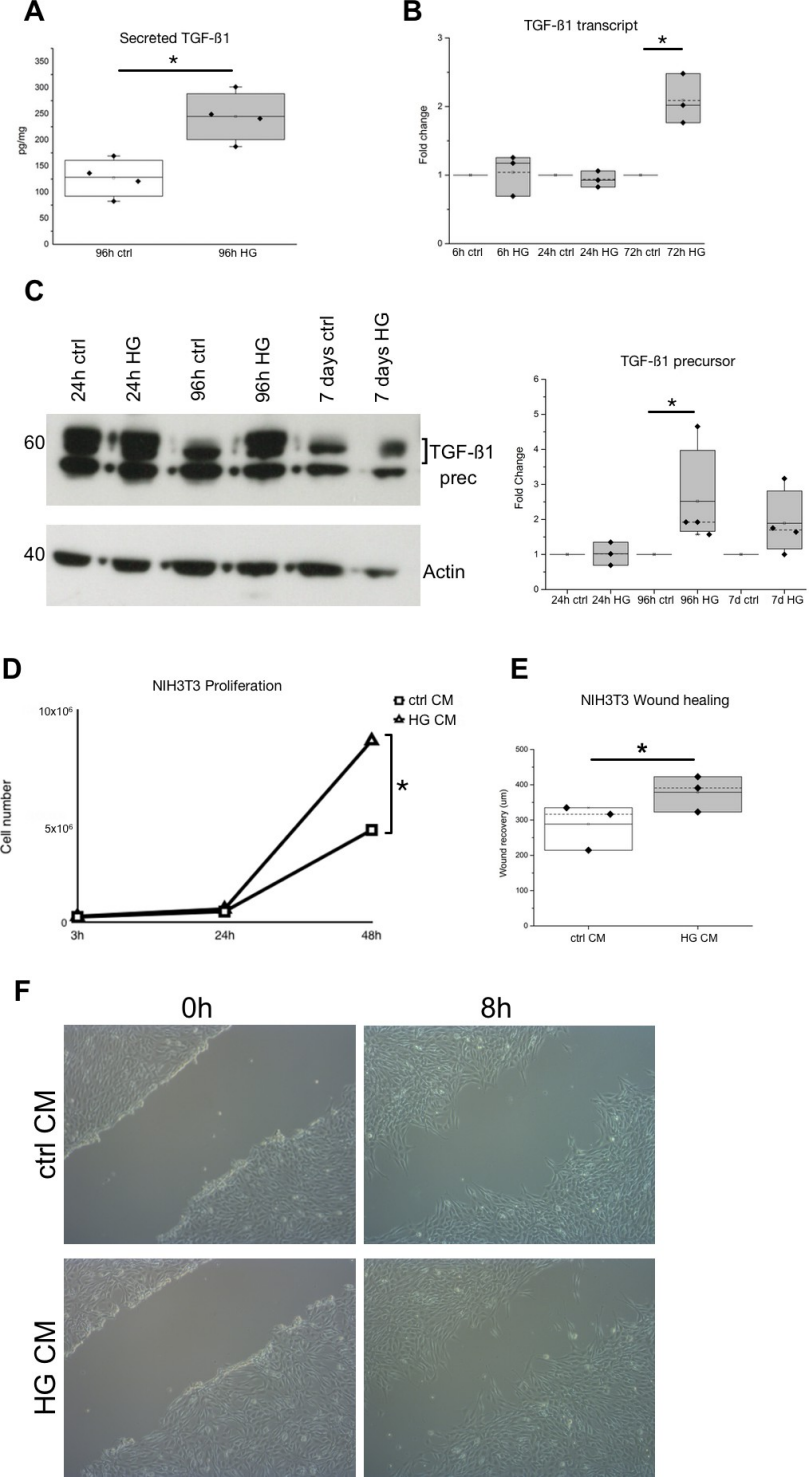
Evaluation of TGF-β1 secretion and expression after HG treatment of primary tubular cell cultures, and effects of conditioned media of HG-treated tubular cells on murine fibroblast proliferation and migration. (**A**) Quantification by ELISA of TGF-β1 secreted in conditioned medium of tubular cells cultured for 96h in control (ctrl) and high glucose (HG) medium. Data are expressed as pg/mg in four independent experiments analysed in duplicate. *p<0.05. (**B**) TGF-β1 transcript expression evaluated by Real-time PCR in primary tubular cell cultures at the indicated time points in control (ctrl) and high glucose (HG) conditions. The relative transcript amounts, calculated as 2^−ΔΔCt^, are represented as fold change with respect to the corresponding control sample considered equal to 1. (n = 3). *p<0.05. (**C**) Representative western blot of TGF-β1 precursor and β-actin proteins evaluated in lysates of primary tubular cell cultures grown for the indicated time points in control (ctrl) and high glucose (HG) medium. In the box plots, the densitometric values of the TGF-β1 precursor bands normalised for corresponding β-actin bands are expressed as fold change with respect to corresponding control samples. (n≥3). *p<0.05. (**D**) Growth curves of NIH3T3 fibroblasts cultured in control conditioned medium (white squares, ctrl CM) and in high glucose conditioned medium (white triangles, HG CM) of primary tubular cell cultures. (n = 3). *p<0.05. (**E**) Wound healing graph representing the recovery of NIH3T3 fibroblasts treated with conditioned medium of primary tubular cell cultures grown for 96h in control (ctrl CM) and HG (HG CM) medium. Data are expressed as μm in three independent experiments. *p<0.05. (**F**) Representative phase contrast images of wound healing assay at the scratch (0h) and after 8h of wound recovering performed in NIH3T3 fibroblasts treated with conditioned medium of primary tubular cultures grown for 96h in control (ctrl CM) and high glucose (HG CM) medium.

We also evaluated the level of TGF-β1, the most prominent fibrogenic cytokine, in the HG-treated tubular cell conditioned media. HG treatment of primary tubular cell cultures induced a significant increase of active TGF-β1 secretion, as evaluated by ELISA assay (Fig 7A). Even the TGF-β1 expression increased, as evaluated by Real-time PCR and western blot (Fig 7B and 7C).

Based on the documented role of TGF-β as major factor driving EMT [31], we have investigated whether the increase of TGF-β1 secretion by our HG-treated tubular cells could activate, by an autocrine mechanism, TGF-β1/Smad signalling in these cells that showed a partial EMT phenotype. We have evaluated the nuclear translocation of Smad2/3 proteins and Smad2 phosphorylation, by western blot (S2A Fig), and the transcript expression of PAI-1, a well-known transcriptional target of TGF-β1/Smad signalling, by Real-Time PCR, (S2B Fig) in control and HG-treated tubular cells, as proxy of TGF-β1/Smad signalling activation [38,39]. The data obtained evidenced that TGF-β1/Smad signalling was not activated in either control or HG-treated cells, suggesting that the increment of TGF-β1 secretion by HG-treated cells did not induce an autocrine activation of TGF-β1/Smad signalling in these cells.

Notably, the proliferation of NIH3T3 murine fibroblasts was significantly increased when cultured in conditioned medium of HG-treated tubular cells (HG CM) as compared with those grown in conditioned medium of control tubular cells (ctrl CM) (Fig 7D). Concomitantly, migration ability was increased in NIH3T3 fibroblasts growth in conditioned medium of HG-treated tubular cells (Fig 7E and 7F).

Globally, these data confirmed that the secretome of tubular cells grown in HG conditions has a pro-inflammatory profile and that HG treatment induced in human tubular primary cell cultures the secretion of cytokines able to induce fibroblast proliferation and migration.

## Discussion

The role of tubular cell EMT in the pathogenesis of renal interstitial fibrosis observed in diabetic nephropathy is still a matter of debate and a conclusive evidence of a full EMT process *in vivo* is still lacking. Although several EMT features have been characterized both in diabetic mouse models and proximal tubular cell lines [10], studies performed in primary tubular cell cultures grown in high glucose conditions are lacking. In fact, at our knowledge, this is the first study investigating the effect of HG on human primary tubular cell cultures. Here, we took advantage of a reproducible and well-characterized cellular model established from human normal cortex specimens and composed of both proximal and distal tubular cells. These primary cell cultures maintained, in the first passages, the phenotypic and molecular properties of the original tissue [22–24], thus studying the HG effect on this *in vitro* model can provide a good representation of *in vivo* tubular response to hyperglycemia. We showed that HG treatment induced only few phenotypic and molecular changes consistent with EMT in these primary cultures. In fact, the cells grown in HG conditions acquired an elongated fibroblast-like morphology, but retained the expression of the epithelial markers Pancytokeratin and Epcam observed in control cultures. Moreover, the proximal tubular cells maintained the co-expression of CD13, N-cadherin and AQ-1 markers, and the distal tubular cells retained the expression of Calbindin and E-cadherin markers even when grown in HG conditions. Notably, unlike tubular cell lines in which N- and E-cadherin were co-expressed [8], we did not observe in our primary tubular cells, not even after HG treatment, a co-expression of N- and E-cadherin, described also *in vivo* as markers of human proximal and distal tubular cells [40]. At the molecular level, our data showed the up-regulation of Snail1, miRNA210 and Vimentin and the increase of thick stress fibers crossing the cytoplasm, all aspects that are consistent with an EMT onset. Notably, the loss of a prominent cortical distribution of stress fibers induced by HG treatment matched with the decrease of N-cadherin expression. This finding is in agreement with the described co-existence of cortical actin distribution and cadherin-based intercellular adhesions [41]. Instead, the expression of Col1a2, Fibronectin and α-SMA, described as fibrogenesis and tubular EMT markers [31], were not increased by HG treatment. Of note, α-SMA protein has never been detected in our cells. At functional level, it has been described that the cells during EMT increased their matrix adhesion and migration [41,42]. Here, we showed that HG decreased the motility of tubular cells without affecting their focal adhesion distribution and adhesion ability.

Overall, the phenotypic and functional features of our HG-treated cells were more suggestive of an activated state of partial EMT rather than an overt EMT phenotype. Notably, the development of an activated cellular state and a partial EMT phenotype, characterized by cells expressing both epithelial and mesenchymal markers, has been also described in other renal fibrosis models [43] and in rat diabetic kidney [13], respectively.

In line with our results, Keller et al. described that primary cultures of human tubular cells treated with TGF-β, the major factor driving EMT [31], showed morphological alterations but maintained a stable expression of E-cadherin, although the expression of Snail1 and Zeb2, known to down-regulate E-cadherin, did not change or even increased [38]. In the same study, TGF-β treatment of human primary tubular cultures increased N-cadherin and Fibronectin expression and activated TGF-β/Smad signalling, unlike what has been observed with HG in our study. These data suggested a different efficacy of TGF-β and HG treatment in inducing tubular EMT, highlighting a higher responsivity of N-cadherin positive proximal tubular cells with respect to E-cadherin positive distal tubular cells towards both treatments.

We also studied the possible role of human tubular cells grown in HG conditions in the onset of a pro-inflammatory and pro-fibrotic microenvironment, by the analysis of control and HG-treated tubular cell secretome coupled with the functional annotation analysis of the corresponding GO-BP terms. This is the first study, to our knowledge, investigating the contribution of tubular cells to cytokine response induced by hyperglycemia in diabetes by using human primary tubular cell cultures grown in high glucose condition. We found that M-CSF, Eotaxin, RANTES, TNF-α and GM-CSF were more up-regulated in conditioned medium of HG-treated tubular cells compared to control. Moreover, the cytokines RANTES and TNF-α that significantly increased at 24h of HG treatment are annotated in GO-BP terms related to “positive regulation of inflammation”. Notably, TNF-α is also found in serum and urine of patients affected by diabetic nephropathy [1]. Conversely, the secretion level of IL-17, IL-2, IL-15, MCP-1, IL-18, IL-9, IL-10, MIP-1a, SDF-1α, IL-12, CTACK, VEGF, LIF and FGF-basic increased significantly less in HG-treated cultures compared to control. Of note, a different secretive behavior was observed for IL-18 in our cellular model compared to HK-2 human proximal tubular cell line, which in HG conditions showed an increment of IL-18 secretion [19]. The presence in our *in vitro* model of both proximal and distal tubular cells might explain this discrepancy and provide a better representation of *in vivo* tubular response to hyperglycemia. Instead, the secretion level of IL-3 and IL-12p40 decreased in HG-treated cultures more than in control cultures and, notably, IL-12p40 together with IL-10 and IL-2 were annotated in GO-BP terms related to a “negative regulation of inflammatory response”.

Thus, the analysis of control and HG-treated tubular cell secretome coupled with the functional annotation analysis of the corresponding GO-BP terms, seems to confirm the idea that HG treatment induces in tubular cells the secretion of cytokines that favor the onset of a pro-inflammatory microenvironment. This inflammatory state, described as able to induce *in vivo* tubular EMT, recruitment of fibrocytes and proliferation/differentiation of fibroblasts to myofibroblasts [2], might be also responsible, likely through an autocrine mechanism, for the partial tubular EMT phenotype observed in our *in vitro* model. On the other hand, the observed HG-dependent increase in TGF-β1 secretion, the major factor driving EMT [31], did not seem to induce an autocrine activation of TGF-β/Smad signalling in our tubular cells. Thus, TGF-β1 did not seem to be responsible *per se* for the observed partial EMT phenotype.

The conditioned medium of our HG-treated tubular cells might also act as a pro-fibrogenic stimulus. In fact, NIH3T3 murine fibroblasts cultured in conditioned media of HG-treated primary tubular cultures showed an increase of proliferation and migration. These findings can be also related to the HG-dependent increment of the expression and secretion of TGF-β1, a well-known inducer of fibroblast proliferation [44], and to the decreased HG-dependent secretion of FGF-basic growth factor, annotated in the “negative regulation of fibroblast migration” GO-BP term. Notably, the tubular cells in HG conditions have been described as involved in the induction of a pro-fibrogenic milieu even *in vivo* [2]. Moreover, previous studies performed in type 1 and 2 diabetic mice showed that treatment with antibodies against TGF-β1 prevented glomerulosclerosis and interstitial fibrosis, thus supporting a critical role for TGF-β1 signalling in the ECM accumulation characterizing diabetic nephropathy.

## Conclusions

In conclusion, our data show that high glucose induces in human tubular primary cell cultures an activated state of partial EMT rather than an overt EMT, and that the secretome of these activated tubular cells has pro-inflammatory properties and is able to induce the proliferation and migration of fibroblasts, supporting an active role of tubular cells in diabetic nephropathy development.

## Supporting information

S1 Fig Phenotypic characterization of HG-treated primary tubular cell cultures. (**A**) Primary tubular cell cultures viability does not change after treatment with control and HG medium. Primary tubular cell cultures grown in control (ctrl) and HG media at the indicated time points were stained with FITC Annexin V/propidium iodide (PI). FACS analysis was performed with MOFLO ASTRIOS instrument and analyzed by Kaluza software (Beckman Coulter, Brea, CA, USA). The acquisition process was stopped when 20,000 events were collected in the population gate. In the representative images, the percentage of viable cells (AnnexinV/PI negative) was reported as mean ± s.d. of three independent experiments. (**B**) High glucose (HG) treatment does not change the percentage of cells expressing epithelial markers in primary tubular cell cultures. Primary tubular cells cultured in control (ctrl) and HG medium for 24h, 96h and 7 days were stained with primary anti-Pancytokeratin (PanCK) or anti-Epcam antibodies and corresponding Alexa conjugated secondary antibodies or with secondary antibodies alone (negative control), and analysed by FACS as previously described. In the representative images the percentage of PanCK- and Epcam-positive cells at 7 days of treatment was reported. (**C**) Stress fiber density is significantly higher in primary tubular cells cultured for 96h in HG media compared to control (ctrl). Stress fiber density analysis has been performed on F-Actin stained cells using ImageJ software and following the Method described in Peacock et al. (Mol. Biol. Cell **18**, 3860–3872, 2007). Briefly, ImageJ Plot Profile function was used to obtain the pixel intensity average (shown as dots in the graph) along lines randomly drawn on cellular cytoplasm in non-overlapping region of fifteen cells randomly chosen in three different fields of a representative control and HG-treated human primary tubular cell culture. (**D**) Evaluation of the adhesion ability of primary tubular cells cultured in control (ctrl) and HG media. The adhesion assay has been performed on 96-well plates as described [28] and dot blot graph shows the absorbance values at 540 nm as obtained at different time points of culture in three independent experiments. (**E**) The percentage of cells positive to the proximal tubular marker CD13 does not change during HG treatment in primary tubular cell cultures. Primary tubular cells cultured in control (ctrl) and HG medium for 24h, 96h and 7 days were stained with anti-CD13 FITC antibody and analysed by FACS as previously described. Percentage of CD13-positive cells was reported as mean ± s.d. of three independent experiments.
(TIF)

S2 Fig Evaluation of TGF-β signalling activation in HG-treated tubular primary cell cultures. (**A**) Representative western blot analysis of Phospho-Smad2 and Smad2/3 protein level in nuclear and cytoplasmic fractions obtained from primary tubular cells cultured in control (ctrl) and HG medium for 24h, 96h and 7 days. The Phospho-Smad2 level and the prevalent cytoplasmic localization of Smad2/3 proteins does not change between control and HG-treated cells at the different time points analysed. Nuclear and cytoplasmic protein fractions of control and HG-treated primary cultures have been obtained as described in Di Stefano et al. [25]. The absence of Tubulin protein band in nuclear fractions and of Histone-H3 protein band in cytoplasmic fractions ensured the quality of sub-fractions obtained. Total homogenate proteins from a Renal Cell Carcinoma cell line, treated with TGF-β1 and with TGF-β1+SB431542 as described [39], are used as positive (ctrl+) and negative (ctrl-) control of TGF-β1 signalling activation, respectively. To detect the specific protein bands, antibodies against Phospho-Ser 465/467 Smad2 (1:1000, Cell Signaling Technology), Total Smad2/3 (1:1000, Cell Signaling Technology), α-Tubulin (1:1000, Cell Signaling Technology) and Histone-H3 (1:4000, clone 96C10, Cell Signaling Technology) have been used. (**B**) PAI-1 transcript expression evaluated by Real-time PCR in primary tubular cell cultures at the indicated time points in control (ctrl) and high glucose (HG) conditions. The expression of PAI-1, a well known transcriptional target of TGF-β/Smad signalling [39], did not significantly increase in HG-treated primary tubular cells. The relative transcript amounts, calculated as 2^−ΔΔCt^, are represented as fold change with respect to the corresponding control sample considered equal to 1. (n = 3). Real-time quantitative PCR assays were carried out, as indicated in the Material and Method section, with the following TaqMan Gene Expression Assays: PAI-1 (Hs01126606_m1) and GAPDH (Hs99998805_m1) (Applied Biosystems) used as housekeeping gene.
(TIF)

S1 File (PDF)
