# Supplementary figures and images for: High glucose induces an activated state of partial epithelial-mesenchymal transition in human primary tubular cell cultures
Source: PLoS One. 2023 Feb 24;18(2):e0279655. doi: 10.1371/journal.pone.0279655 (PMC9956654; doi:10.1371/journal.pone.0279655)

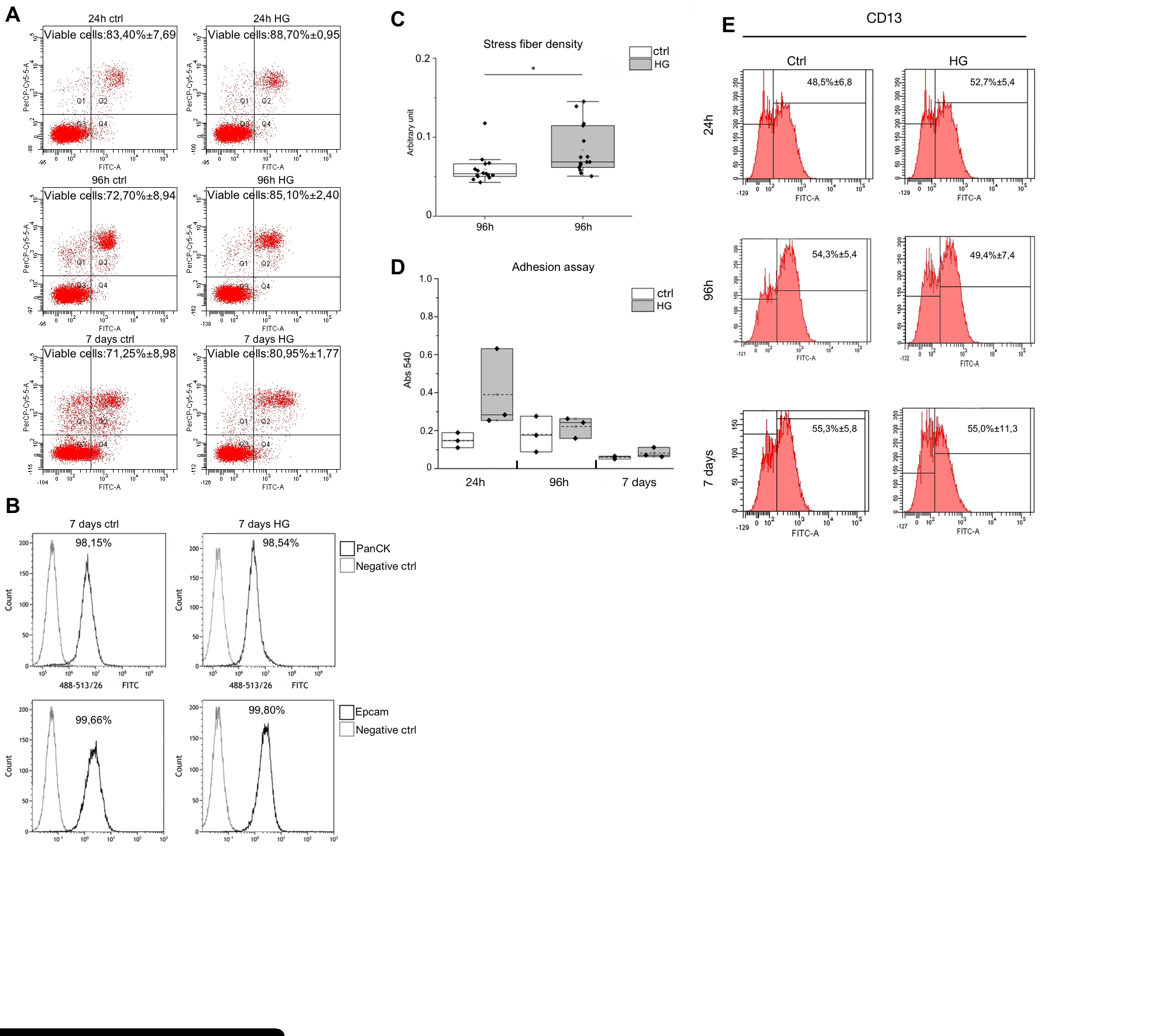

Supplement: S1 Fig — (A) Primary tubular cell cultures viability does not change after treatment with control and HG medium. Primary tubular cell cultures grown in control (ctrl) and HG media at the indicated time points were stained with FITC Annexin V/propidium iodide (PI). FACS analysis was performed with MOFLO ASTRIOS instrument and analyzed by Kaluza software (Beckman Coulter, Brea, CA, USA). The acquisition process was stopped when 20,000 events were collected in the population gate. In the representative images, the percentage of viable cells (AnnexinV/PI negative) was reported as mean ± s.d. of three independent experiments. (B) High glucose (HG) treatment does not change the percentage of cells expressing epithelial markers in primary tubular cell cultures. Primary tubular cells cultured in control (ctrl) and HG medium for 24h, 96h and 7 days were stained with primary anti-Pancytokeratin (PanCK) or anti-Epcam antibodies and corresponding Alexa conjugated secondary antibodies or with secondary antibodies alone (negative control), and analysed by FACS as previously described. In the representative images the percentage of PanCK- and Epcam-positive cells at 7 days of treatment was reported. (C) Stress fiber density is significantly higher in primary tubular cells cultured for 96h in HG media compared to control (ctrl). Stress fiber density analysis has been performed on F-Actin stained cells using ImageJ software and following the Method described in Peacock et al. (Mol. Biol. Cell 18, 3860–3872, 2007). Briefly, ImageJ Plot Profile function was used to obtain the pixel intensity average (shown as dots in the graph) along lines randomly drawn on cellular cytoplasm in non-overlapping region of fifteen cells randomly chosen in three different fields of a representative control and HG-treated human primary tubular cell culture. (D) Evaluation of the adhesion ability of primary tubular cells cultured in control (ctrl) and HG media. The adhesion assay has been performed on 96-well plate [file pone.0279655.s001.tif]

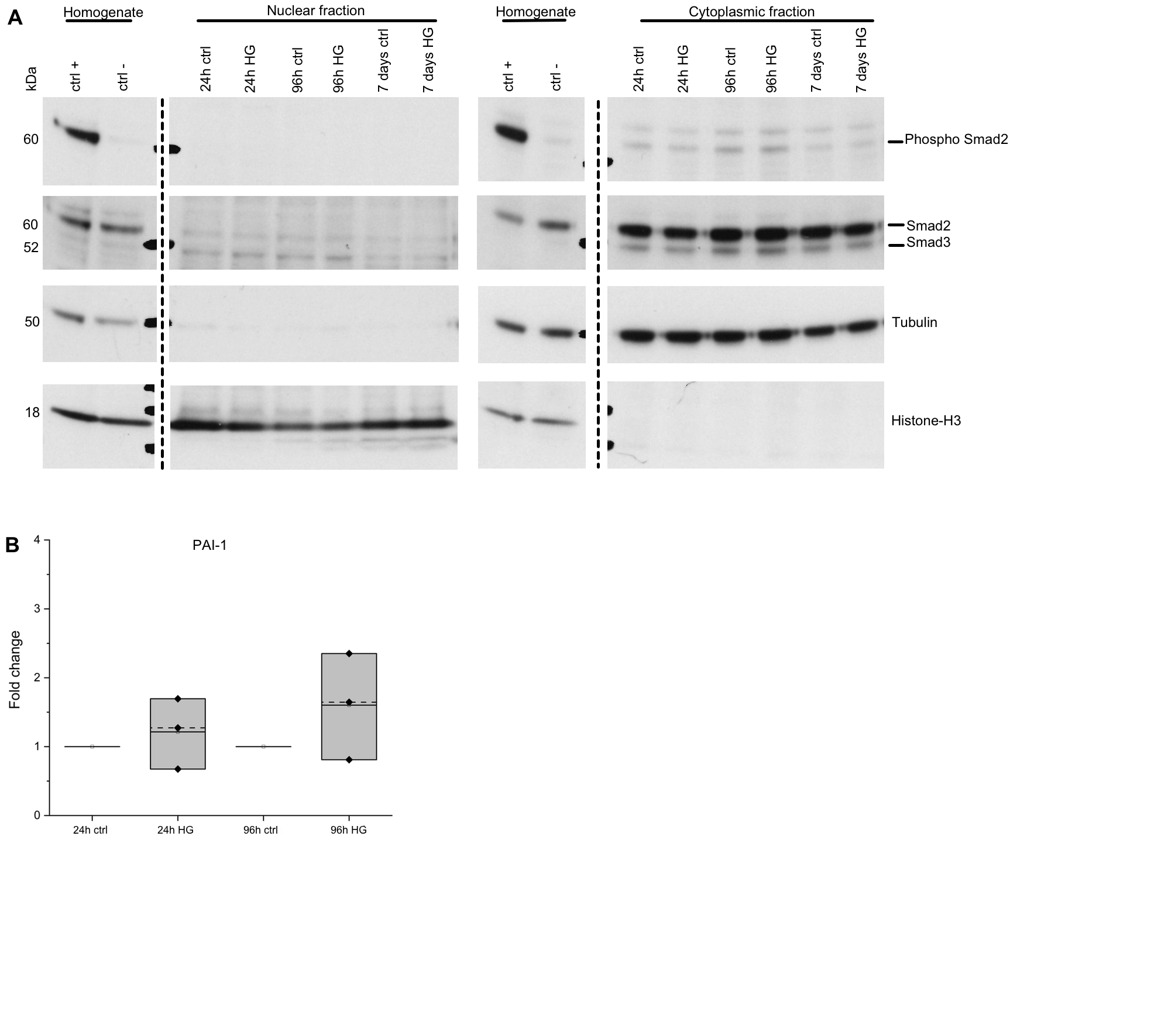

Supplement: S2 Fig — (A) Representative western blot analysis of Phospho-Smad2 and Smad2/3 protein level in nuclear and cytoplasmic fractions obtained from primary tubular cells cultured in control (ctrl) and HG medium for 24h, 96h and 7 days. The Phospho-Smad2 level and the prevalent cytoplasmic localization of Smad2/3 proteins does not change between control and HG-treated cells at the different time points analysed. Nuclear and cytoplasmic protein fractions of control and HG-treated primary cultures have been obtained as described in Di Stefano et al. [25]. The absence of Tubulin protein band in nuclear fractions and of Histone-H3 protein band in cytoplasmic fractions ensured the quality of sub-fractions obtained. Total homogenate proteins from a Renal Cell Carcinoma cell line, treated with TGF-β1 and with TGF-β1+SB431542 as described [39], are used as positive (ctrl+) and negative (ctrl-) control of TGF-β1 signalling activation, respectively. To detect the specific protein bands, antibodies against Phospho-Ser 465/467 Smad2 (1:1000, Cell Signaling Technology), Total Smad2/3 (1:1000, Cell Signaling Technology), α-Tubulin (1:1000, Cell Signaling Technology) and Histone-H3 (1:4000, clone 96C10, Cell Signaling Technology) have been used. (B) PAI-1 transcript expression evaluated by Real-time PCR in primary tubular cell cultures at the indicated time points in control (ctrl) and high glucose (HG) conditions. The expression of PAI-1, a well known transcriptional target of TGF-β/Smad signalling [39], did not significantly increase in HG-treated primary tubular cells. The relative transcript amounts, calculated as 2−ΔΔCt, are represented as fold change with respect to the corresponding control sample considered equal to 1. (n = 3). Real-time quantitative PCR assays were carried out, as indicated in the Material and Method section, with the following TaqMan Gene Expression Assays: PAI-1 (Hs01126606_m1) and GAPDH (Hs99998805_m1) (Applied Biosystems) used as housekeeping gene. (TIF) [file pone.0279655.s002.tif]
