# Supplementary material for: High glucose induces an activated state of partial epithelial-mesenchymal transition in human primary tubular cell cultures
Source: PLoS One. 2023 Feb 24;18(2):e0279655. doi: 10.1371/journal.pone.0279655 (PMC9956654; doi:10.1371/journal.pone.0279655)
Supplement: S1 File — (PDF) [file pone.0279655.s003.pdf]

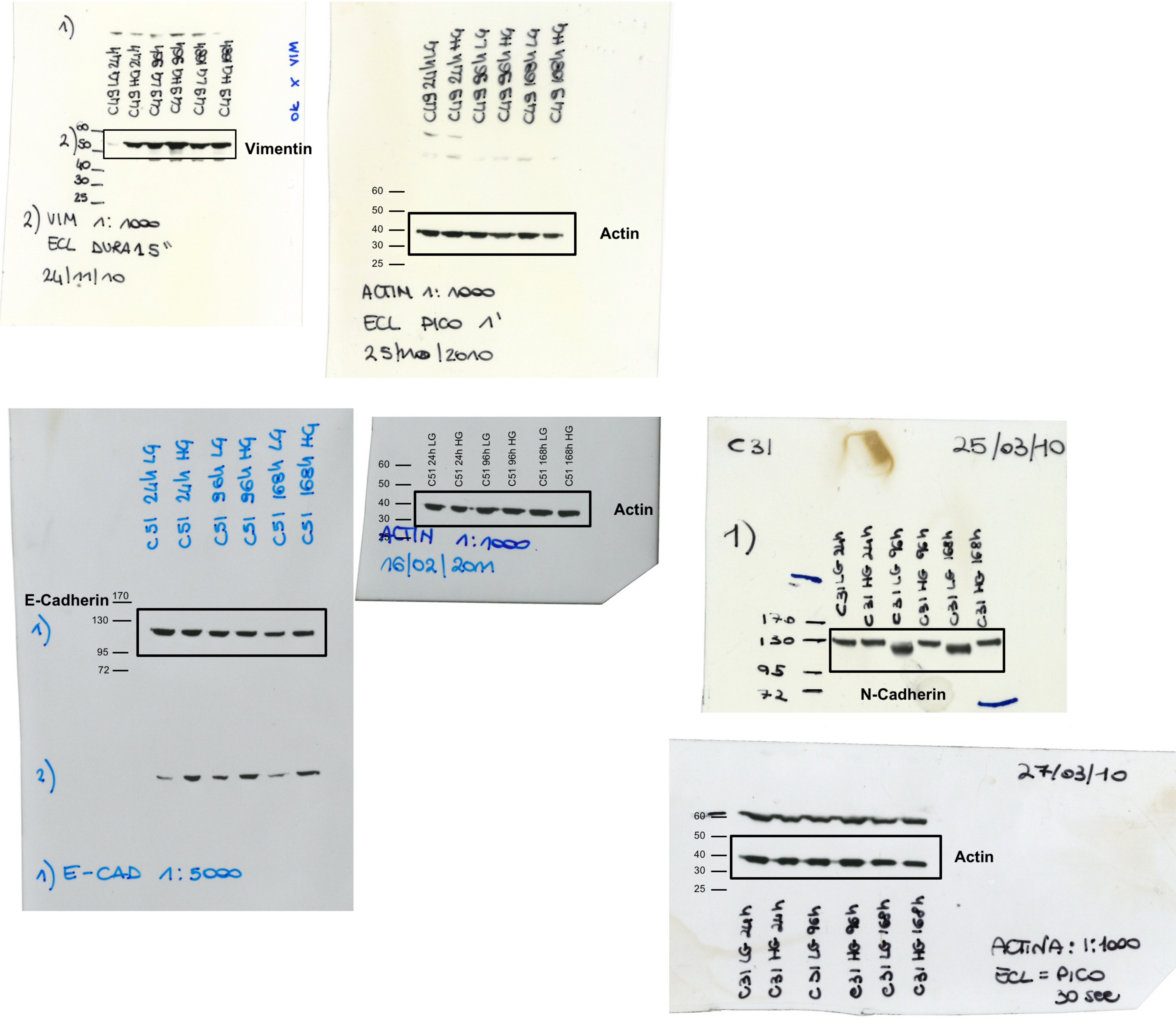

Uncropped Western Blots of Figures 5C

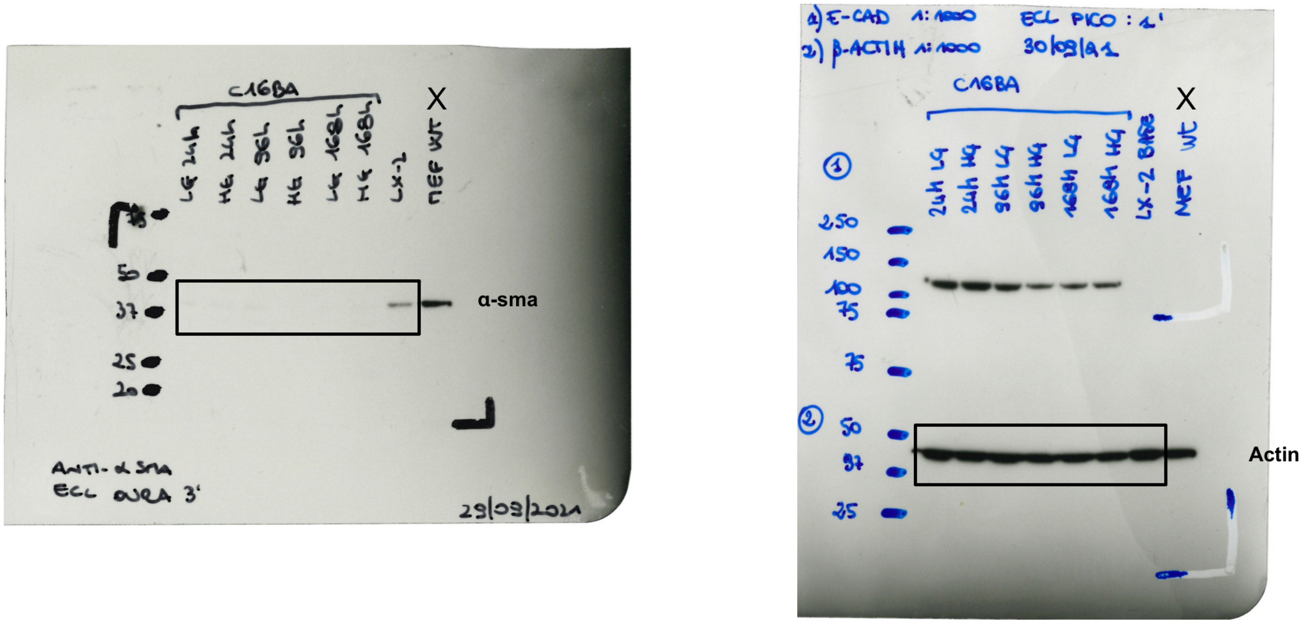

Uncropped Western Blots of Figure 5D

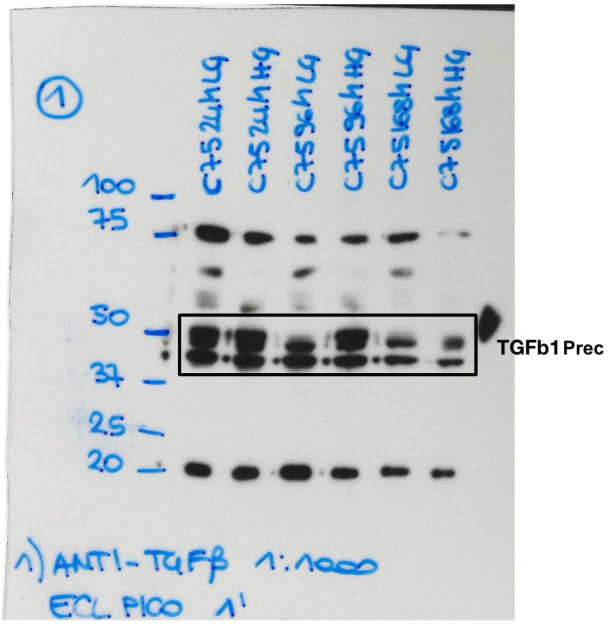

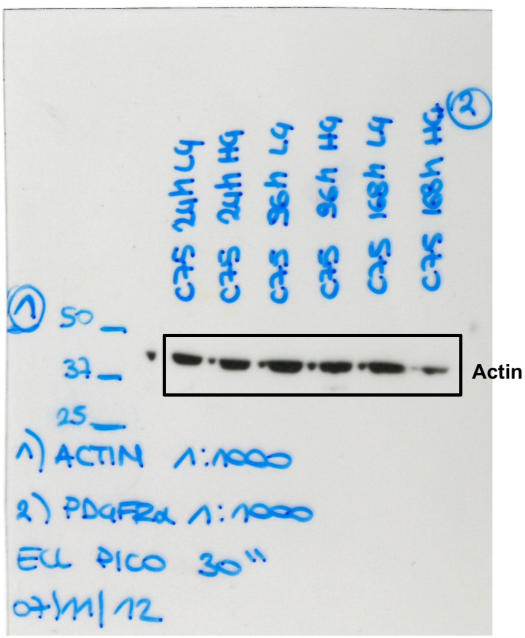

Uncropped Western Blots of Figure 7C

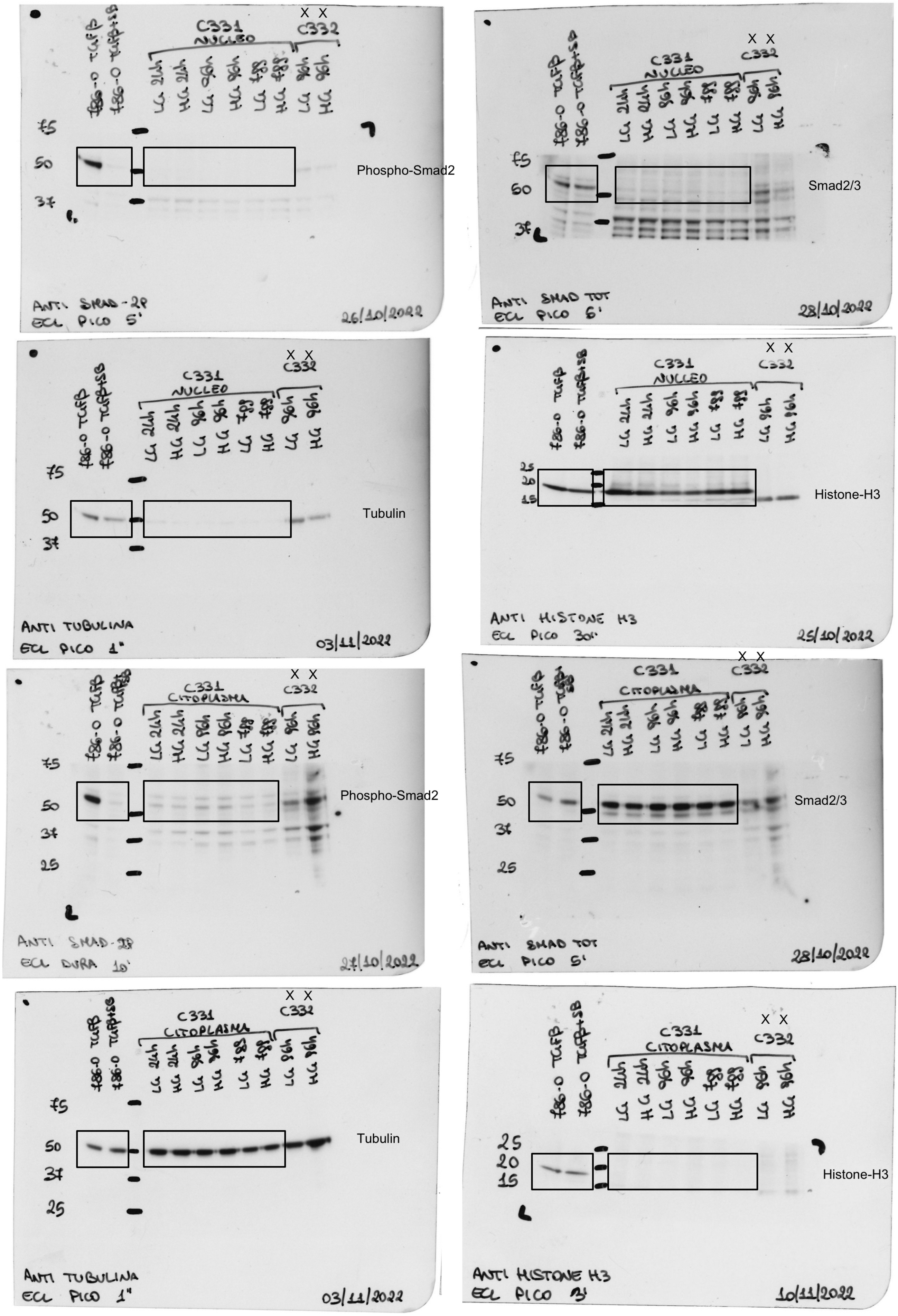

Uncropped Western Blots of Figure S2A

All the films have been captured using Kyocera-TASKalfa Scanner at 300 dpi of resolution
